# Multiparametric imaging for detection and characterization of hepatocellular carcinoma using gadoxetic acid-enhanced MRI and perfusion-CT: which parameters work best?

**DOI:** 10.1186/s40644-017-0121-9

**Published:** 2017-06-28

**Authors:** Mustafa Kurucay, Christopher Kloth, Sascha Kaufmann, Konstantin Nikolaou, Hans Bösmüller, Marius Horger, Wolfgang M. Thaiss

**Affiliations:** 1 Department of Radiology, Diagnostic and Interventional Radiology, Eberhard Karls University, Hoppe-Seyler-Str. 3, D-72076 Tuebingen, Germany; 2 Department of Pathology, Eberhard Karls University, Liebermeisterstraße 8, D-72076 Tuebingen, Germany

**Keywords:** Hepatocellular Carcinoma, MRI, perfusion-CT, contrast agent, perfusion

## Abstract

**Background:**

MRI and perfusion-CT (PCT) are both useful imaging techniques for detection and characterization of liver lesions. The aim of this study was to compare the diagnostic accuracy of imaging parameters derived from PCT and gadoxetic acid-enhanced MRI in patients with hepatocellular carcinoma (HCC).

**Methods:**

36 patients with liver cirrhosis and a total of 67 lesions referred to our hospital for multi-parametric diagnosis of HCC-suspected liver lesions in the setting of liver cirrhosis were prospectively enrolled and underwent PCT and MRI. HCC diagnosis was confirmed either by histology (*n* = 60) or interval growth (*n* = 7). For PCT, mean/max blood flow (BF), blood volume (BV), k-trans, arterial liver perfusion (ALP), portal venous perfusion (PVP) and hepatic perfusion index (HPI) were quantified. Two readers identified the lesions based on single maps each being blinded to the number of lesions.

MRI-protocol included fat-suppressed T1w-VIBE sequences obtained before, 2, 5, 10 and 20 min after the injection of gadoxetic acid as well as non-enhanced coronal HASTE, axial T1w-VIBE, fat-suppressed T2w-TSE and DWI. Quantitative analysis was performed using enhancement ratios between tumor and liver parenchyma for post-contrast in the hepatobiliary phase (RIR_HB_), arterial (ER_a_) and late-venous (ER_v_) phases as well as signal intensity ratios (liver/parenchyma) on T1w (RIR_T1_) and T2w (RIR_T2_).

**Results:**

In PCT analysis, all lesions exhibited high BF_max_ values (63–250 mL/100 g tissue) and were visible on HPI maps with high degrees of arterial blood supply of (HPI > 96%).

In MRI, RIR_HB_ was negative in 8/67. 12/67 HCCs were missed on DWI. 46/67 HCCs showed wash-in and 47/67 HCC showed wash-out of contrast agent. 6/67 HCCs were missed on T1w and 11/67 were missed on T2w-sequences when analyzed separately, while analysis of multiparametric MRI combining typical enhancement pattern, visibility on hepatobiliary phase and T1w-images the same number of lesions as PCT irrespective of their size (1–19 cm) were detected. Quantification of early enhancement by ER_a_ or ER_v_ did not improve detection rates.

**Conclusions:**

Perfusion-CT and gadoxetic acid-enhanced MRI were comparable in detecting HCC lesions. For PCT a mean HPI > 96% proved to be a very robust parameter for detection and characterization of HCC.

## Background

Finding the best suited imaging technique for detection and characterization of hepatic lesions on the background of cirrhotic liver is still a challenge [1, 2]. Current guidelines recommend the use of dynamic contrast-enhanced CT (also volume perfusion CT, PCT) or MRI studies for assessment of “typical” enhancement patterns such as wash-in and wash-out [3]. These features reflect the temporal differences in the arterial and portal-venous blood supply of liver lesions vs. liver parenchyma. Increasing arterial blood supply is expected during dedifferentiation of hepatocytes (HCC-precursors) along the pathway of hepato-carcinogenesis [4]. However, liver cirrhosis might as well lead to an increase in arterial supply of the liver parenchyma due to architectural distortion and subsequent portal hypertension. This in turn may limit the contrast differences between HCC-precursors (e.g. dysplastic nodules) and liver parenchyma, hampering their accurate detection. With increasing dedifferentiation of focal liver lesions, however, their arterial supply steadily increases and thus, the wash-in effect becomes more obvious.

The opposite lesion-to-parenchyma-contrast is expected on the late venous enhancement phases where mainly or exclusively arterially supplied liver lesions show wash-out of contrasted blood compared to liver cells that still enhance due to their predominantly portal-venous supply [5]. The documentation of these enhancement patterns requires repeated measurements of the liver (3–4 post-contrast phases) on both CT and MRI [6]. Generally, fixed delay times are used for arterial, portal-venous and late venous or equilibrium phase in order to standardize liver imaging. However, tumor-dependent characteristics as well as differences in individual blood circulation may lead to inadequate selection of the time points for enhancement documentation. Moreover, MRI sequences can be time consuming, so that comparison with CT-enhancement phases may be limited. MRI uses additional information for both detection and characterization of liver lesions e.g. ancillary findings like T1w-signal intensity, T2w-signal intensity on unenhanced studies, new functional information derived from diffusion-weighted imaging (DWI) and in particular the use of liver specific contrast agents [7, 8]. The latter has advanced to a widely recommended imaging technique based on its metabolic information with respect to the degree of enhancement in the late hepatobiliary phase [9]. This phase helps for better detection of even smaller lesions [10] and also for characterization of HCC-precursors which are expected to take up this contrast agent to different degrees compared to normal liver tissue and thus serve as a discriminator between HCC and its predecessors [11].

PCT on the other hand represents a development of conventional multi-phase-CT paired with sophisticated software post-processing capable of separating the arterial from the portal-venous blood supply to both liver lesions and liver parenchyma. Based on this differentiation, detection and characterization of focal lesions can be markedly improved.

The purpose of this study was to compare the diagnostic accuracy of gadoxetic acid-enhanced MRI and liver perfusion-CT derived imaging parameters for both detection and characterization of HCC using both qualitative and quantitative measurements.

## Methods

An institutional review board–approved HIPAA-compliant prospective study was performed, and the requirement for informed consent was waived. From March 2008 through September 2016, 36 cirrhotic patients (29 men; mean age 62.6 years) with 67 lesions were enrolled and all gave their written informed consent. All patients were referred to our hospital for complementary imaging diagnosis of HCC-suspicious liver lesions diagnosed offsite by either CT, contrast-enhanced ultrasound or MRI.

Inclusion criteria for this study were liver cirrhosis with biopsy (*n* = 60) confirmed HCC and timely closely (less than 50 days) performed dual PCT and MR imaging or alternatively interval growth/new occurrence of HCC-suspicious liver lesions (*n* = 7). Exclusion criteria were impaired renal function, known allergic diathesis to iodized contrast agents, contraindications to MRI like claustrophobia, implanted pacemakers or refusal to participate in this study.

All patients had confirmed liver cirrhosis. Underlying cause of cirrhosis were hepatitis C virus infection [HCV] (*n* = 12); hepatitis B virus infection [HBV] (*n* = 4); alcohol abuse (*n* = 11); combination of viral hepatitis + alcohol abuse (*n* = 2); cryptogenic cirrhosis (*n* = 5); chronic cholangitis (*n* = 1) and hemochromatosis (*n* = 1).

### MR imaging technique

MRI was performed using a 1.5-T magnet (*n* = 33 examinations, Aera or Avanto, Siemens Healthineers, Forchheim, Germany) or a 3-T magnet (*n* = 34 examinations, Skyra, Siemens Healthineers, Forchheim, Germany) with a phased-array coil for signal reception. Sequences were similar across platforms but were optimized for each scanner. A minimum of the following sequences were performed in all patients before intravenous (i.v.) injection of contrast material: axial non-contrast GRE T1-weighted, axial respiratory-gated T2-weighted (TSE), DWI (b, 0/400/800 s/mm^2^). Subsequently a fat-suppressed T1w-VIBE sequence was obtained before i.v., contrast agent administration. Afterwards, application of 0.25 μmol/kg gadolinium-based hepatocellular contrast agent (gadoxetic acid, Primovist, Bayer Schering Pharma, Berlin, Germany) were administered i.v. followed by 20 ml of sterile saline solution were administered at a 2 mL/s rate using a power injector. Four fat-suppressed T1w-VIBE sequences were applied after contrast injection using three contrast-enhanced series with delay times of each 35–45 s in-between up to 5 s and then 10s and 20s later. Slice thickness varied among the sequences being 5–6 mm with 10% gap. Matrix size varied between 256 × 128 and 320 × 150. The field of view was adapted to the patient size (250–350 mm).

### Perfusion-CT (PCT) imaging technique

All PCT examinations were performed on a 128-row CT scanner (Somatom Definition Flash or Force, Siemens Healthineers, Forchheim, Germany). The CT protocol consisted of a non-enhanced abdominal low-dose CT which was obtained to localize the liver porta. Subsequently, a scan range between 11.4–17.6 cm z-axis coverage was planned over the liver, followed by perfusion-CT of the tumor (PCT) using adaptive spiral scanning technique. Perfusion parameters were: 80 kV, 100mAs, collimation 64 × 0.6 mm with z-flying focal spot and 26 CT repeated scans of the entire liver tumor within a total scan time of 40s. During perfusion scanning, the patients were asked to resume shallow breathing for the entire duration of the study. In case of respiratory shortness, oxygen was provided to the patients and abdominal belts were applied for motion reduction of the anterior abdominal wall. 50 ml Ultravist 370 (Bayer Vital, Leverkusen, Germany) was injected at a flow rate of 5 mL/s in an antecubital vein followed by a saline flush of 50 ml NaCl at 5 mL/s, and a start delay of 7 s. From the PCT raw data, axial images with a slice thickness of 3 mm for perfusion analysis were reconstructed without overlap, using a smooth tissue convolution kernel (B10f). All images were transferred to an external workstation (Multi-Modality Workplace, Siemens) for analysis*.*

Radiation dose for PCT depends on the scan length ranging between 9 mSv and 14 mSv for a scan length of 11.4 cm and 17.6 cm, respectively.

### Reading

Two readers with 4-years and >20-years of experience in reading oncological CTs and MRIs analyzed all datasets. Readers were aware of the presence of HCC suspected lesions but blinded to number and size in all examinations. All available MRI sequences and perfusion maps from PCT were analyzed. MRI reading was first performed separately for each sequence with the purpose of assessing their contribution for lesion detection and characterization based on signal features whereas the final MRI-detection rate was judged based on the sum of all MRI-derived information. In 7 cases a consensus between both readers was achieved after initial discrepancies (4 on DWI reading, 3 on T1w reading). When HCC were not visible on T1w images the lesion size measurement was obtained on the available sequence that delineated the lesion.

### Quantitative measurements in MRI

We measured lesion diameter by determining the largest diameter of the mass. After i.v. injection of gadoxetic acid, the following quantitative parameters were evaluated: the enhancement ratio (ER) equivalent to (post-contrast signal intensity (SI) – pre-contrast SI/pre-contrast SI) × 100 in the arterial post-contrast phase (ERa) and portal-venous phase (ERv) [12].

For DWI, the average numerical maps were calculated using large regions of interest (ROIs, 10 s/mm^2^).

The SI of the largest representative enhancing ROI in the lesion and of one adjacent liver parenchyma ROI were also collected in the unenhanced T1w-sequence and T2w-sequence. The relative intensity ratio (RIR) between lesion and liver parenchyma was calculated on pre-contrast T1w-images (RIR_T1_) and T2w-images (RIR_T2_) equivalent to SInod/SIpar, where SInod is the SI of the nodule, and SIpar is the SI of the liver parenchyma [12]. Additionally, the RIR was calculated for the hepatobiliary phase (RIR_HB_) using the same formula.

Moreover, we drew regions of interest in the paraspinal musculature to evaluate potential influences of the two different field strengths (1.5 T and 3 T) on signal intensity and corresponding tissue ratios.

### Qualitative image analysis

The presence of contrast wash-in on arterial phase images and wash-out on late venous phase images (up to 5 s post-injection) was registered on dynamic MR-images.

### Quantitative measurements with PCT

Motion correction, noise reduction, and threshold-based exclusion of bone, fat and air was performed with Syngo Volume Perfusion CT Body (Siemens Healthineers, Forchheim, Germany). A ROI was drawn at the maximal lesion outline in perfusion maps from PCT and the diameter of the lesion in cm was documented.

From PCT, blood flow (BF; mL/100 mL/min), blood volume (BV; mL/100 mL), arterial liver perfusion (ALP; mL/100 mL/min), portal venous perfusion (PVP; mL/100 mL/min) and hepatic perfusion index (HPI; %) were determined. The deconvolution model was used for BF, BV and k-trans measurements whereas the maximum slope model was used for ALP, PVP and the calculated HPI index.

### Statistical analysis

SPSS (Version 22, IBM, Armonk, NY, USA) was used for statistical analysis. All values are expressed as mean ± standard deviation. Pearson’s r was used for correlation analysis. Unpaired t-test was used for group comparison.

## Results

MRI and PCT were performed within a mean of 8.8 days (SD 21.4); all examinations could be included in the following analysis. A total of 67 lesions were identified using all available MRI sequences or PCT. Diagnosis was confirmed with histology or growth at follow up. ADC maps were not diagnostic in 3/67 cases. In one patient, no ERa/ER late could be calculated.

### Quantitative and qualitative measurements in MRI

First, MRI data were analyzed and the mean HCC lesion size measured on T1w MRI images was 5.12 cm (SD 4.06). 6/67 HCCs were missed on *T1w-images*. Mean lesion size in the subgroup where lesions were missed on T1w-images was 3.3 cm (SD 1.6) compared with 5.3 mm (SD 4.1) in the subgroup that correctly detected liver lesions on T1w-images.

59/67 HCC lesions were visible on *hepatobiliary phase images*. Mean RIR_HB_ was 0.69 (SD 0.26). The mean RIR_HB_ of visible lesions was 0.65 (SD 0.23) compared to 1.0 (SD 0.2) in lesions that were not visible (*p* < 0.0001). None of the RIR_HB_ negative cases showed wash-out. A summary of the measured parameters of the RIR_HB_ negative cases is given in Table 1.

**Table 1:** MRI data for negative cases in hepatobiliary phase

	Negative cases / mean (±SD)	Positive cases/mean (±SD)	*p* values	case#1	case#2	case#3	case#4	case#5	case#6	case#7	case#8
Visible in hepatobiliary phase	0/8	59/59		0	0	0	0	0	0	0	0
Visible in T1w	7/8	54/59		1	1	1	1	1	1	1	0
Visible in t2w	7/8	49/59		1	1	0	1	1	1	1	1
Visible in DWI	6/8	50/59		1	1	0	0	1	1	1	1
Wash-in visible	4/8	53/59		1	0	0	0	0	1	1	1
Wash-out visible	0/8	47/59		0	0	0	0	0	0	0	0
Size in cm	3.28(±1.70)	5.43(±4.25)	n.s.	1.50	5.00	3.50	2.20	1.00	2.90	6.50	3.60
T1 RIR	1.17(±0.21)	0.82(±0.23)	0.0002	1.31	1.20	1.31	1.11	1.00	1.55	1.04	0.82
T2 fs RIR	1.30(±0.45)	1.95(±0.94)	n.s.	1.71	1.28	0.76	0.89	0.74	1.26	1.72	2.00
ADC	0.87(±0.14)	0.89(±0.29)	n.s.	0.87	0.52	0.95	0.88	0.92	0.98	0.87	0.95
ERa	50.13(±26.58)	84.30(±38.59)	0.0198	75.97	11.52	11.69	65.88	33.67	84.62	67.68	50.00
ER late	44.68(±25.99)	57.27(±33.13)	n.s.	39.86	8.76	5.66	54.71	41.71	90.23	60.95	55.56
RIR HB	1.00(±0.19)	0.65(±0.23)	0.0001	1.08	0.98	0.68	1.02	0.99	1.42	0.94	0.92

Hepatobiliary negative cases are shown in column 1 in comparison to hepatobiliary positive cases in column 2. Individual values for negative cases#1–8 are shown on the right. Quantitative values are given as mean ± standard deviation (SD). *P* values are given for group comparison. RIR relative enhancement ratio. ADC apparent diffusion coefficient. ER enhancement ratio

Mean overall *RIR*_*T1*_ was 0.86 (SD 0.25). Mean RIR_T1_ in the subgroup where the lesions were missed was 0.89 (SD 0.18) as compared with the subgroup of detected liver lesions on T1w where the RIR_T1_ was 0.86 (SD 0.26, n.s.). Only one of the “T1-negative” cases was detectable on T2w images. Additional information in such lesions was needed, as all of the lesions showed wash-in and 5/6 lesions showed wash-out.

On fat-saturated T2w-images 11/67 were missed on qualitative reading. Mean *RIR*_*T2*_ was 1.87 (SD 0.92). Mean RIR_T2_ in the subgroup where the lesions were missed was 1.16 (SD 0.36) as compared with the subgroup of detected liver lesions on T2w where the RIR_T2_ was 2.0 (SD 0.94, *p* < 0.005).

Using ROC characteristics, RIR_T2_ with a cut-off value of >1.5 resulted in a sensitivity/specificity of 71.4%/81.8%. This would result in 2 additional positive cases that were not detected visually. However, only 40/56 of the visual positive cases fulfill these criteria.

Next, *diffusion weighted images* were assessed. Mean ADC was 0.89 (SD 0.28) s/mm^2^. 12/67 HCCs were missed by *DWI*. 17 lesions showed no restriction in diffusion.

The presence or absence of wash-in and wash-out was analyzed both qualitatively and quantitatively. 46/67 HCCs showed *wash-in* whereas 47/67 HCC showed *wash-out* enhancement pattern. Mean ERa was 80.16 (SD 38.97) whereas mean ERv was 55.75 (SD 32.61). There was a significant difference between the mean ER_a_ in lesions with wash-in (87.15 SD 37.39) and lesions without wash-in (41.00 SD 24.22) (*p* = 0.0004). No significant difference between the mean ER_v_ in lesions with or without wash-out.

The combined information from several MRI sequences led to identification of all lesions. The typical enhancement pattern with wash-in and wash-out was present in 47/67 lesions. With the addition of visibility in the hepatobiliary phase, 59/67 lesions were identified. With the addition of T1w images, all lesions could be identified as such.

We calculated ratios for non-hepatic reference tissue (paraspinal muscle) in non-enhanced sequences, arterial and hepatobiliary phase. There were no significant differences between the ratios calculated for examinations at 1.5 T and 3 T (1.5 T arterial/unenhanced: 1.05 (± 0.09), 3 T: 1.07 (± 0.12), *p* = 0.55 n.s.; 1.5 T late phase/unenhanced: 1.13 (± 0.06), 3 T 1.17 (± 0.11), *p* = 0.1). Also, the enhancement ratio for the paraspinal muscle did not vary significantly (1.5 T: 8.85 (± 7.45), 3 T 12.45 (± 10.18), *p* = 0.25).

**Fig. 1:**
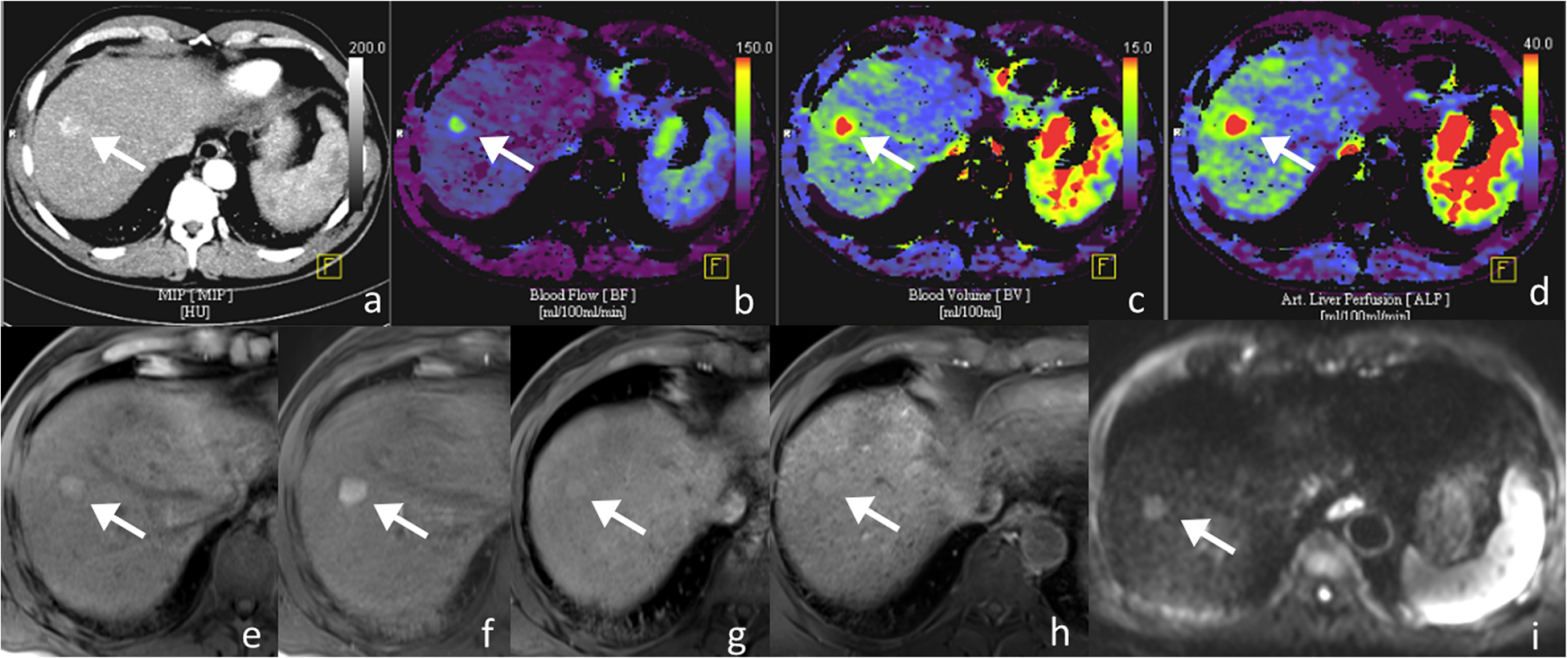
64-year-old male patient with 2.5 cm large HCC in liver segment 8 (arrows). Maximum intensity projection (**a**) and perfusion-CT (PCT) blood flow (**b**), blood volume (**c**) and arterial liver perfusion (**d**) color-coded maps are shown. Signal intensity on unenhanced fat-saturated (fs) T1w (**e**) is hyperintense with typical wash-in enhancement pattern on post-contrast fs T1w in the arterial (**f**), phase. No contrast wash-out is seen in the late post-contrast phase (**g**) whereas in the hepatobiliary (**h**) phase the tumor stays isointense to the background liver parenchyma. The tumor was hyperintense on T2w (not shown) including DWI (**i**, T2-shine through effect), but there was no restriction of water diffusivity. Calculated ER_a_ was 117.8. On PCT the HPI was 100%, BF_max_ = 250.9 mL/100 g tissue; BV = 13.1 mL/100 g tissue; ALP = 35.3 mL/100 g tissue. Notably, despite exclusive arterial supply of the histologically proven tumor, there was no wash-out in the late venous phase (**h**) and also poor delineation of the tumor in the hepatobiliary phase (**g**)

A patient example is shown in Fig. 1.

### Quantitative measurements with PCT

All 67 HCC lesions were identified on perfusion maps. The mean lesion size measured on PCT maps was 5.17 cm (SD 4.09); no significant difference was observed when compared to MRI measurements. All analyzed lesions were arterialized on perfusion maps with an average BF of 97.34 mL/100 mL/min (SD 45.14), BV of 19.78 mL/100 mL (SD 10.05) and an HPI of 96.4% (SD 8.18). An overview of additional PCT results is given in Table 2.

**Table 2:** Overview of HCC perfusion-CT data

	Mean	SD
BF max	139.55	(±69.77)
BF avg	97.34	(±45.14)
BV max	19.16	(±11.63)
BV avg	19.78	(±10.05)
ALP max	68.77	(±29.48)
ALP avg	52.80	(±22.62)
PVP max	0.71	(±2.85)
PVP avg	1.98	(±4.64)
k-trans max	38.30	(±32.17)
k-trans avg	36.33	(±28.61)
HPI max	98.82	(±4.59)
HPI avg	96.40	(±8.18)

Maximum and average values (max and avg) are given as mean ± standard deviation (SD) for the following parameters: BF blood flow. BV blood volume. ALP arterial liver perfusion. PVP portal venous perfusion. k-trans permeability surface area product. HPI hepatic perfusion index

### Correlations between gadoxetic acid-enhanced MRI and PCT

To identify redundant parameters obtained with MRI and PCT, we performed correlation analysis between the quantitative values. ER_a_ correlated with BF_max_ and BF_avg_ (*r* = 0.278, *p* = 0.023; *r* = 0.353, *p* = 0.003), k-trans_avg_. And k-trans_max_ (*r* = 0.292, *p* = 0.017; *r* = 0.330, *p* = 0.006) and with ALP_avg_ and ALP_max_ (*r* = 0.289, *p* = 0.018; *r* = 0.367, *p* = 0.004). ERv showed no correlation with PCT-based perfusion parameters.

HPI correlated moderately or weak with the presence of wash-in and wash-out on MRI (*r* = 0.46 and 0.34, *p* < 0.001 and *p* = 0.003, respectively). When comparing the HPI values from lesions that were wash-in positive vs. wash-in negative, the mean HPI for the wash-in positive lesions on MRI was 97.73 (SD 5.40) compared to 86.81 (SD 15.60) in the lesions that were wash-in negative (*p* < 0.001). RIR_HB_ inversely correlated with HIP (*r* = −0.27, *p* = 0.026).

## Discussion

Even though multi-parametric MRI is an excellent tool, and is known to be superior to CT due to its better tissue contrast as well as lack of radiation, additional imaging tools to accommodate those patients that either don’t want or cannot have an MRI are mandatory. For this reason, we aimed at comparing the performance of perfusion-CT with that of gadoxetic acid-enhanced MRI using both qualitative and quantitative measures for detection and characterization (imaging signatures) of hepatocellular carcinoma in this study.

With respect to *detectability* of HCC, our results show equal rates for gadoxetic acid-enhanced MRI and PCT in cirrhotic livers. In PCT, all lesions showed high degrees of arterialization with a mean HPI >96% and were therefore well-demarcated from the surrounding liver parenchyma on the ALP and HPI color-coded maps which have been primarily used for lesion identification.

With MRI, only the combined use of image information derived from all applied sequences could compete with PCT for lesions detection. The great heterogeneity of image characteristics registered throughout all used MRI sequences in our HCC-cohort did not allow to identify a reliable combination of pulse sequences or parameters, which could make a multi-sequence approach superfluous. Complementarity of MR image features was unpredictable. For example, only one “T1w-negative” tumor could be additionally detected on T2w images demonstrating that even combinations of few imaging parameters often fail. In order to understand why some of these tumors could not be identified on T2w-images, we additionally used intensity ratios (RIR_T2_) to quantify the magnitude of signal intensity differences between T2w-detected and T2w-overlooked tumors. Although there was a significant difference between detected and non-detected tumors for the whole cohort, the use of cut-off values for RIR_T2_ only minimally improved the sensitivity with a significant number of cases that would have been missed.

Even the use of intensity ratios for the hepatobiliary phase (RIR_HB_) showed several limitations as 8/67 tumors were missed. Our results are in line with previous publications on MRI that describe the challenges for HCC characterization with gadoxetic acid. Nonetheless, negative results in the hepatobiliary phase, are well known to be beneficial for non-invasive assessment of tumor differentiation. Several authors discussed the lack of standardization of post-contrast delayed imaging. Zhang et al. found the early-post contrast phase to be superior compared to delayed phase [13].

In terms of *characterization*, MRI and perfusion-CT showed differences. Hence, considering the degree of arterial blood supply of a hepatic lesion as indicative for the presence of a HCC as demonstrated in this study by PCT and also by previously reported study results is essential. Perfusion-CT offers robust and accurate values for HPI that homogenously characterize all tumors [14–16]. Based on the same principle, the currently used enhancement patterns like the presence of wash-in and wash-out proved to be less sensitive for lesion identification based on differences in the contribution of arterial and portal-venous dual blood supply to the liver and tumor and seem to be in part subjective. For improved detectability of these patterns, enhancement ratios have been proposed for both the arterial and portal-venous perfusion phases [12]. At this point, our data is in line with that of previous reports validating the benefit of using these ratios [12]. Nonetheless, even the use of enhancement ratios could not entirely enforce their weighting as unfaultable perfusion parameters. Semi-quantification of early arterial supply to the liver lesions proved more accurate with ERa lying >1 in all cases, but many of them showed low levels of arterialization which explains why they were in part missed by visual assessment. Moreover, the time point of assessment during dynamic contrast enhanced MRI depends on the length of measurements for each sequence (phase) which are significantly longer compared to PCT. This explains why correlations of PCT-parameters like the magnitude of blood flow (BF) and/or blood volume (BV) with the qualitative and semi-quantitative MRI measurements of enhancement ratios were limited. Another potential cause of poor arterial phase imaging (wash-in) is the so-called transient severe motion (TSM) artifact which has been reported in around 6% [17]. However, TSM was not encountered in our series.

Weak correlations were found for ERa with mean and max BF and BV which are parameters directly reflecting tumor vascularization and in particular with the arterial liver perfusion (ALP) which represents the ratio of arterial blood supply to the tumor. Correlations between ERv and the amount of portal-venous perfusion (PVP) were even not significant. Expectedly, the intensity ratios in the hepatobiliary (RIR_HB_) phase showed inverse correlation with the hepatic perfusion index (HPI) which represents the percentage of ALP/ALP + PVP (×100). Hence, as the contrast between lesion and liver parenchyma increases as the tumor becomes more and more arterially supplied the contrast ratio on gadoxetic acid-enhanced hepatobiliary phase steadily decreases as there is no uptake by the tumor cells.

The additional use of DWI was of limited value both due to negative results or false-positive due to T2-shine through effects.

PCT non-invasively enabled confirmation of HCC-diagnosis based on the high percentage of arterial supply (mean HPI > 96%). This approach seems to be reliable, easily accomplished and practicable in the routine work-up of hepatic nodules in cirrhotic livers. Nonetheless, a small percentage of HCCs might present as hypo-vascular lesions and thus challenge the radiologist in terms of vascularization-based characterization. However, separate calculations for the arterial and portal-venous tumor supply using PCT seem to permit differentiation of these tumors from other HCC-precursor lesions by showing that they are mostly or entirely arterially supplied [18]. Hence, such cases show very high HPI levels irrespective of the magnitude of BF. Lesion hyper- or isointensity in the hepatobiliary phase of gadoxetic acid is generally associated with benign behavior, in particular in smaller lesions. However, contradictory reports also exist [19, 20].

PCT is a relatively new non-invasive imaging technique which delivers similar information as the classical CT hepatic arteriography (CTHA) + CT arterial portography (CTAP) that was used for a long time as a gold-standard for HCC-diagnostic [16] comparable even with histology [21]. Due to its invasiveness, this latter technique has been abandoned in the last decade and has been recently successfully substituted by PCT which delivers comparable results but in a non-invasive fashion. Previous reports on the superiority of MRI, in particular gadoxetic acid-enhanced, over multi-phase CT (MDCT) are in our opinion explained by the limitation of exam protocols of MDCT leading in part to an equitation of inadequate enhancement phases (e.g. due to co-existing morbidities like cardiac failure, lowered circulation time, etc.) as well as in part to the lack of perfusion quantification and separate calculation of the degree of tumor arterialization which are essential for tumor detection and characterization [22–25]. Earlier reports comparing CTHA/CTAP with MDCT have shown that lesion detection and characterization could thus be significantly increased over MDCT in particular if using double phase CTHA [26, 27]. Imai et al. reported similar results for CTHA/CTAP as compared with multimodality HCC-imaging using MDCT and SPIO (small particles iron oxide)-enhanced MRI [28]. The additive benefit of CTHA/CTAP together with Gd-DTPA-enhanced dynamic studies over Gd-DTPA enhanced MRI together with SPIO-enhanced MRI was repeatedly reported [28]. Yim et al. recommended the combined use of gadoxetic acid-enhanced MRI with CTHA/AP for further increase in diagnostic accuracy in particular for HCCs <1 cm [21, 29]. They explained differences in detection and characterization of small HCC by the higher degree of arterial enhancement achieved with CTHA/CTAP compared to gadoxetic acid-enhanced MRI. Compared to CTHA/CTAP, our PCT-protocol consists of up to 26 consecutive arterial phases increasing the detectability of tumor arterialization. While lesion detection was equal for both techniques (PCT and gadoxetic acid-enhanced MRI) with the multitude of MRI-derived parameters always compensating the weakness of every single sequence analyzed separately for itself, in terms of characterization MRI proved less persuasive. As we did not find very strong correlations between the different parameters from the two modalities, complementary rather than redundant values in terms of lesion characterization seem to be present. Current guidelines on this diagnostic field even recommend the use of combined modality imaging for HCC-confirmation [3]. In our opinion, the only limitation for the broad use of larger scan-length PCT is dose exposure which restrains its repeated use e.g. for monitoring purposes. Nevertheless, PCT is more robust in patients with pronounced perihepatic ascites, which can limit the MRI signal from the liver substantially.

Our study has some limitations. First, MRI data was acquired using different scanners and slightly different protocols due to the long recruitment period, however, we did not observe any influence on the calculated ratios and used uninvolved reference tissue. Second, there were no other liver lesions (HCC-precursors) included. Third, special software is a prerequisite for tumor quantification using PCT. However, to our knowledge dedicated software programs are offered by most vendors.

## Conclusions

Perfusion-CT and gadoxetic acid-enhanced MRI were comparable in detecting HCC lesions. PCT represents an equal imaging tool to accommodate those patients that are not amenable for MRI.

## Abbreviations

ALP: Arterial liver perfusion
BF: Blood flow
BV: Blood volume
DWI: diffusion-weighted imaging
ER_a_: Arterial enhancement ratio
ER_v_: Venous enhancement ratio
Gadoxetic acid: Gadolinium ethoxybenzyl diethylenetriamine pentaacetic acid
k-trans: Permeability surface area product, or k-trans
PCT: perfusion CT
PVP: Portal venous perfusion
RIR_HB_: Relative intensity ratio hepato-biliary phase
RIR_T1_: Relative intensity ratio on T1w images
ROI: Region of interest
SD: Standard deviations
